# Clinical Characteristics of Disseminated Strongyloidiasis, Japan, 1975–2017

**DOI:** 10.3201/eid2603.190571

**Published:** 2020-03

**Authors:** Mitsuru Mukaigawara, Masashi Narita, Soichi Shiiki, Yoshihiro Takayama, Shunichi Takakura, Tomokazu Kishaba

**Affiliations:** Harvard Kennedy School, Cambridge, Massachusetts, USA (M. Mukaigawara);; Okinawa Chubu Hospital, Uruma, Okinawa, Japan (M. Mukaigawara, M. Narita, S. Shiiki, Y. Takayama, S. Takakura, T. Kishaba)

**Keywords:** dissemination, strongyloidiasis, human T-cell lymphotrophic virus type 1, HTLV-1, meningitis, Strongyloides stercoralis, parasites, nematodes, roundworms, Japan

## Abstract

This condition has clinical phenotypes based on severity; identification of occult dissemination with neurologic manifestations is lifesaving.

## Medscape CME ACTIVITY

In support of improving patient care, this activity has been planned and implemented by Medscape, LLC and Emerging Infectious Diseases. Medscape, LLC is jointly accredited by the Accreditation Council for Continuing Medical Education (ACCME), the Accreditation Council for Pharmacy Education (ACPE), and the American Nurses Credentialing Center (ANCC), to provide continuing education for the healthcare team.

Medscape, LLC designates this Journal-based CME activity for a maximum of 1.00 ***AMA PRA Category 1 Credit(s)*™**. Physicians should claim only the credit commensurate with the extent of their participation in the activity.

Successful completion of this CME activity, which includes participation in the evaluation component, enables the participant to earn up to 1.0 MOC points in the American Board of Internal Medicine's (ABIM) Maintenance of Certification (MOC) program. Participants will earn MOC points equivalent to the amount of CME credits claimed for the activity. It is the CME activity provider's responsibility to submit participant completion information to ACCME for the purpose of granting ABIM MOC credit.

All other clinicians completing this activity will be issued a certificate of participation. To participate in this journal CME activity: (1) review the learning objectives and author disclosures; (2) study the education content; (3) take the post-test with a 75% minimum passing score and complete the evaluation at http://www.medscape.org/journal/eid; and (4) view/print certificate. 

### Release date: February 19, 2020; Expiration date: February 19, 2021

### Learning Objectives

Upon completion of this activity, participants will be able to:

- Describe classification of disseminated strongyloidiasis into 3 clinical phenotypes, according to a case series
- Determine clinical and laboratory findings in disseminated strongyloidiasis, according to a case series
- Identify treatment of disseminated strongyloidiasis, according to a case series

### CME Editor

**Jude Rutledge, BA,** Technical Writer/Editor, Emerging Infectious Diseases. *Disclosure: Jude Rutledge has disclosed no relevant financial relationships.*

### CME Author

**Laurie Barclay, MD,** freelance writer and reviewer, Medscape, LLC. *Disclosure: Laurie Barclay, MD, has disclosed no relevant financial relationships.*

### Authors

*Disclosures:* ***Mitsuru Mukaigawara, MD; Masashi Narita, MD; Soichi Shiiki, MD; Yoshihiro Takayama, MD; Shunichi Takakura, MD;*** *and* ***Tomokazu Kishaba, MD, have*** *disclosed no relevant financial relationships.*
