# Supplementary material for: Clinical Characteristics of Disseminated Strongyloidiasis, Japan, 1975–2017
Source: Emerg Infect Dis. 2020 Mar;26(3):401–8. doi: 10.3201/eid2603.190571 (PMC7045830; doi:10.3201/eid2603.190571)
Supplement: Related texts

# Review article

## Abstract

Clinical characteristics of disseminated strongyloidiasis, the severest form of strongyloidiasis, are not well described. We conducted a retrospective, consecutive chart review of patients with disseminated strongyloidiasis admitted to Okinawa Chubu Hospital in Okinawa, Japan, during January 1975–December 2017. The 70 patients were classified into 3 clinical phenotypes: dissemination (32 patients [45.7%]), occult dissemination with meningitis caused by enteric organisms (12 patients [17.1%]), and occult dissemination with culture-negative suppurative meningitis (26 patients [37.1%]). Associated mortality rates were 56.3%, 16.7%, and 11.5%, respectively, and sepsis occurred in 40.6%, 58.3%, and 11.5% of cases, respectively. Common symptoms included fever (52.9% of patients), headache (32.9%), and altered mental status (24.3%). Patients were treated with thiabendazole (before 2003) or ivermectin (after 2003). Our findings show that disseminated strongyloidiasis has clinical phenotypes in terms of severity and that identification of occult dissemination, a mild form with prominent neurologic manifestations, is lifesaving.

Strongyloidiasis is a nematode infection caused by *Strongyloides stercoralis*, a parasitic roundworm*.* Parasites of the genus *S. stercoralis* are characteristic for their ability to replicate within hosts (*1*). Patients have clinical characteristics of systemic strongyloidiasis when their immune status is impaired (*2*), such as through the use of steroids (*3*), medication associated with organ transplantation (*4*), and initiation of chemotherapy (*5*). Strongyloidiasis has been prevalent in the tropics and subtropics and is reemerging as a critical disease in the immunocompromised patient population (*6*).

The life cycle of the *S. stercoralis* roundworm consists of external and autoinfective cycles (*7*). Infective filariform larvae penetrate the skin, enter the venous circulation, and migrate to the pulmonary circulation. The larvae are then swallowed and remain in the small intestine to mature into the parthenogenic female adult *S. stercoralis* worm (*8*). The noninfectious rhabditiform offspring can develop into infective filariform larvae while still in the intestine and reinfect the host. These autoinfective filariform larvae enable the unique autoinfective life cycle of *S. stercoralis* parasites to continue indefinitely in humans.

When the host immune status is compromised, the autoinfective cycle can be enhanced, enabling increasing numbers of autoinfective larvae to disseminate throughout the body. The clinical complications of disseminated strongyloidiasis are a result of the increasing numbers of autoinfective larvae traveling through the body, with many larvae carrying enteric bacteria from the intestine (*9*). Penetrated larvae carry enteric organisms to multiple organ systems, causing sepsis (*10*–*12*), pneumonia (*10*,*11*,*13*), and meningitis (*10*,*11*,*14*–*18*).

Early recognition of dissemination is essential, yet little has been known about its clinical, laboratory, and microbiological characteristics (*1*). Our study aimed to provide characteristics of patients with disseminated strongyloidiasis. We conducted a retrospective, consecutive chart review of patients diagnosed with disseminated strongyloidiasis in an area of human T-cell lymphotrophic virus type 1 (HTLV-1) infection endemicity in Japan during January 1975–December 2017.

## Methods

### Study Setting

This study was conducted at Okinawa Chubu Hospital in Okinawa, Japan, which has a subtropical climate; strongyloidiasis is endemic to Okinawa (*19*). The region also has a high prevalence of HTLV-1 infection (*20*). Okinawa Chubu Hospital is one of the largest teaching hospitals in Okinawa.

### Study Design and Patients

We consecutively reviewed the charts of adult patients (>18 years of age) diagnosed with disseminated strongyloidiasis during January 1975–December 2017 and admitted to Okinawa Chubu Hospital. We classified disseminated strongyloidiasis into 3 clinical phenotypes: dissemination, occult dissemination with meningitis caused by enteric organisms (*14*), and occult dissemination with culture-negative suppurative meningitis (*15*).

We defined dissemination as filariform larvae detected from specimens other than the gastrointestinal tract (i.e., feces, duodenal fluid, and gastric juice). We defined occult dissemination with meningitis caused by enteric organisms as meningitis caused by enteric organisms combined rhabditiform larvae detected from the gastrointestinal tract (e.g., in feces and gastric juice); enteric organisms were *Bacteroides* spp., *Enterococcus* spp., *Escherichia coli*, *Enterobacter* spp., *Klebsiella* spp., *Bifidobacterium* spp., *Clostridium perfringens*, *Proteus mirabilis*, *Streptococcus gallolyticus* (*bovis* group), and *Campylobacter* spp. (*21*). We defined occult dissemination with culture-negative suppurative meningitis as cerebrospinal fluid (CSF) culture-negative meningitis with CSF neutrophil-dominant pleocytosis of >500 cells/mm^3^ (*16*) combined with rhabditiform larvae detected from the gastrointestinal tract (e.g., in feces and gastric juice). 

Patients with nosocomial meningitis and meningitis patients with prior receipt of antibiotics (within 7 days of performing lumbar puncture) were excluded from the analysis. We performed parasitologic examinations by direct microscopic examinations, formalin–ether concentration technique, or both interchangeably on the basis of clinical symptoms.

### Ethics Statement

We collected demographic data, clinical characteristics, clinical diagnosis, and microbiologic data for all patients in a secure database after identifying information of included patients was removed. This study was approved by the Institutional Review Board of Okinawa Chubu Hospital (approval no. H29–75).

## Results

### Patient Population

We identified 70 patients during the study period (Table 1); 42 (60%) were men. Median age of patients was 61 years (range 21–96 years); all patients were born before 1964. Thirty-two patients (32/70 [45.7%]) had dissemination, 12 (12/70 [17.1%]) had occult dissemination with meningitis caused by enteric organisms, and 26 (26/70 [37.1%]) had occult dissemination with culture-negative suppurative meningitis. Thirty-six tested patients (36/39 [92.3%]) had positive serologic assay results for HTLV-1. Six patients (6/70 [8.6%]) had solid organ malignancy, 5.7% (4/70) had diabetes mellitus, and 2.9% (2/70) had cirrhosis. Six patients (8.6%) regularly used steroids; doses were equivalent to 5–30 mg of daily prednisolone. The mortality rate was highest in the dissemination group (18/32 [56.3%]), followed by the occult dissemination with meningitis caused by enteric organisms (2/12 [16.7%]) group and the occult dissemination with culture-negative suppurative meningitis (3/26 [11.5%]) group.

**Table 1:** Demographic characteristics of patients with disseminated strongyloidiasis, Okinawa Chubu Hospital, Uruma, Japan, 1975–2017*

Characteristic	All patients		Dissemination		Occult dissemination with meningitis/enteric organisms		Occult dissemination with culture-negative suppurative meningitis	
No. patients	70/70 (100)		32/70 (45.7)		12/70 (17.1)		26/70 (37.1)	
Sex								
M	42 (60)		19 (59.4)		6 (50)		17 (65.3)	
F	28 (40)		13 (40.6)		6 (50)		9 (34.7)	
Median age, y (range)	61 (21–96)		67 (25–96)		47 (21–84)		57 (38–92)	
Immunocompromised status								
HTLV-1 infection†	36/40 (90.0)		17/20 (85.0)		8/9 (88.9)		11/11 (100)	
Steroid use	6 (8.6)		5 (15.6)		1 (8.3)		0 (0)	
Solid organ malignancy	6 (8.6)		4 (12.5)		2 (16.7)		0 (0)	
Diabetes mellitus	4 (5.7)		4 (12.5)		0 (0)		0 (0)	
Cirrhosis	2 (2.9)		2 (6.3)		0 (0)		0 (0)	
Death	23 (32.9)		18 (56.3)		2 (16.7)		3 (11.5)	

*Values are no. (%) unless indicated. HTLV-1, human T-cell lymphotrophic virus type 1. †Serologic test for HTLV-1 infection was not available until the 1980s.

The number of annually reported cases was decreasing during the study period. The maximum number of annual cases was 7 (Figure). Most strongyloidiasis cases in the last decade of the study period were classified as dissemination.

**Figure:** Number of reported cases of disseminated strongyloidiasis, by clinical phenotype, Okinawa Chubu Hospital, Uruma, Japan, 1975–2017.

### Clinical Manifestations

Common symptoms included fever (37/70 [52.9%] patients), headache (23/70 [32.9%]), altered mental status (17/70 [24.3%]), and nausea/vomiting (10/70 [14.3%]) (Table 2). In terms of laboratory findings, the average absolute eosinophil count was 225 cells/µL (range 0–1,482 cells/µL).

**Table 2:** Symptoms and clinical diagnoses of patients with disseminated strongyloidiasis, Okinawa Chubu Hospital, Uruma, Japan, 1975–2017

Characteristic	No. patients							
	All patients		Dissemination		Occult dissemination with meningitis/enteric organisms		Occult dissemination with culture-negative suppurative meningitis	
No. patients	70 (100)		32 (45.7)		12 (17.1)		26 (37.1)	
Symptoms*								
Fever	37 (52.9)		15 (46.9)		8 (66.7)		14 (53.8)	
Headache	23 (32.9)		3 (9.4)		7 (58.3)		13 (50.0)	
Altered mental status	17 (24.3)		7 (21.9)		3 (25.0)		7 (26.9)	
Nausea or vomiting	10 (14.3)		4 (12.5)		1 (8.3)		5 (19.2)	
Abdominal pain	6 (8.6)		2 (6.3)		2 (16.7)		2 (7.7)	
Anorexia	5 (7.1)		4 (12.5)		1 (8.3)		0 (0)	
Constipation	1 (1.4)		0 (0)		1 (8.3)		0 (0)	
Diarrhea	1 (1.4)		0 (0)		0 (0)		1 (3.8)	
Clinical diagnosis†								
Full-blown dissemination‡	9 (12.9)		9 (28.1)		0 (0)		0 (0)	
Sepsis	23 (32.9)		13 (40.6)		7 (58.3)		3 (11.5)	
Meningitis (enteric)	22 (31.4)		10 (31.3)		12 (100)		0 (0)	
Meningitis (culture-negative)	34 (48.6)		8 (25)		0 (0)		26 (100)	
Pneumonia	27 (38.6)		26 (81.3)		0 (0)		1 (3.8)	
Paralytic ileus	17 (24.3)		15 (46.9)		1 (8.3)		1 (3.8)	

*Other symptoms that were only noted in patients with dissemination included cough (2 cases), and rash, fatigue, convulsion, and syncope (1 case each). †Other diagnoses that were only identified in patients with dissemination included peritonitis (6 cases), dermatitis (5 cases), and esophagitis (1 case). ‡Full-blown dissemination is the severest form of dissemination and hyperinfection, referring to patients with paralytic ileus and pneumonia caused by enteric organisms, as well as either sepsis or meningitis caused by enteric organisms.

The main clinical diagnoses included meningitis, pneumonia, sepsis, and paralytic ileus (Table 2). Twenty-three patients (32.9%) had clinical manifestations of sepsis caused by enteric organisms, 22 patients (31.4%) had clinical manifestations of meningitis caused by enteric organisms, and 34 patients (48.6%) had clinical manifestations of culture-negative suppurative meningitis. Nine patients had highly critical conditions with evidence of multiorgan involvement, including the gastrointestinal (paralytic ileus), pulmonary (pneumonia caused by enteric organisms), central nervous (meningitis), and circulatory (sepsis caused by enteric organisms) systems. The mortality rate was 77.8% (7/9) in these patients. Such clinical features (*22*) have been referred to as “full-blown” dissemination at our institution (*10*). 

One case-patient with full-blown dissemination, a woman in her 60s, had fever, vomiting, headache, and altered mental status. On arrival to the hospital, the patient was obtunded, and neck stiffness was noted. The results of lumbar puncture were suggestive of bacterial meningitis caused by gram-negative bacilli. On direct microscopic examination, multiple larvae were identified in feces, sputum, CSF, ascites, and gastric juice. The patient’s condition deteriorated despite treatment for both strongyloidiasis and bacterial meningitis. Her clinical course also was complicated by paralytic ileus and bacterial pneumonia caused by *K. pneumoniae*. Gram staining of CSF was notable for gram-negative bacilli, and both blood and CSF cultures yielded *E. coli*. The patient died on hospital day 11. On autopsy, larvae were identified in the skin and lungs (*11*).

The clinical course of patients with occult dissemination with bacterial meningitis caused by enteric organisms was less severe than full-blown dissemination, yet remained critical without appropriate treatment. One such patient, a man in his 50s, sought care at our emergency department in the 1980s for a 1-day history of headache, vomiting, shaking, and chills. On examination, the patient had neck stiffness. Results of lumbar puncture were notable for a white blood cell count of 4,104 cells/µL (77% of polymorphonuclear neutrophils). Larvae were detected in feces. The patient was started on thiabendazole, chloramphenicol, and ampicillin. Gram staining of CSF samples was negative, but culture later yielded *E. coli*. Blood culture obtained on admission was negative. The patient responded well to the treatment, and larvae were no longer detected in feces by hospital day 6 (*14*).

Occult dissemination with culture-negative suppurative meningitis occurred in less critical conditions. One case-patient, a woman in her 90s, was brought to our hospital in the 1980s for altered mental status and chills. The patient was obtunded, and results of CSF analysis was consistent with suppurative meningitis (white blood cell count of 3,812/cells/µL [73% of polymorphonuclear neutrophils]). Gram staining of CSF was negative for organisms, and culture results were sterile. Parasitologic examination was positive only in feces. The patient responded well to antibiotics and thiabendazole and was discharged to home on hospital day 9 (*15*).

### Parasitologic and Microbiologic Results

Larvae were identified from samples including stool (from 60/70 [85.7%] patients), sputum (30/70 [42.9%]), gastric juice (24/70 [34.3%]), ascites (4/70 [5.7%]), urine (3/70 [4.3%]), CSF (2/70 [2.9%]), and biopsy specimens (Table 3). No antibody testing or PCR was used in making a diagnosis, except for 1 patient who had necrotizing esophagitis (*23*).

**Table 3:** Parasitologic investigation and culture results of patients with disseminated strongyloidiasis, Okinawa Chubu Hospital, Uruma, Japan, 1975–2017*

Characteristic	No. (%) patients							
	All patients		Dissemination		Occult dissemination with meningitis/enteric organisms		Occult dissemination with culture-negative suppurative meningitis	
No. patients	70 (100)		32 (45.7)		12 (17.1)		26 (37.1)	
Specimens with larvae†								
Stool	60 (85.7)		22 (68.8)		12 (100)		26 (100)	
Sputum	30 (42.9)		30 (93.8)		0 (0)		0 (0)	
Gastric juice	24 (34.3)		16 (50)		3 (25)		5 (19.2)	
Blood culture results‡								
* Escherichia coli*	6 (8.6)		4 (12.5)		2 (16.7)		0 (0)	
* Klebsiella pneumoniae*	7 (10.0)		4 (12.5)		1 (8.3)		2 (7.7)	
* Streptococcus gallolyticus*	5 (7.1)		1 (3.1)		3 (25)		1 (3.8)	
CSF culture results‡								
* E. coli*	9/65 (13.8)		4/27 (14.8)		5 (41.7)		0 (0)	
* K. pneumoniae*	6/65 (9.2)		3/27 (11.1)		3 (25)		0 (0)	
* S. gallolyticus*	5/65 (7.7)		1/27 (3.7)		4 (33.3)		0 (0)	

*CSF, cerebrospinal fluid. †In dissemination, larvae also were detected from ascites (4 cases), urine (3 cases), CSF (2 cases), and skin, bronchoalveolar lavage, and biopsies of the lung, duodenum, and liver (1 case each). ‡In dissemination, blood cultures also were positive for *Enterobacter aerogenes*, *Enterococcus* spp., and *S. infantarius* (1 case each), and CSF cultures were positive for *E. aerogenes* and *Streptococcus* spp. (1 case each). In occult dissemination with meningitis (enteric organisms), blood cultures also were positive for *E. agglomerans* (1 case), and CSF cultures were positive for *Enterococcus* spp. (1 case).

*E. coli*, *K. pneumoniae*, and *S. gallolyticus* were the most common organisms detected from blood and CSF cultures, causing 7%–10% of bacteremia. Other organisms that caused bacteremia included *E. aerogenes*, *S. infantarius*, and *Enterococcus* spp.

### Treatment

Before 2002, patients were treated with thiabendazole. Thiabendazole was administered at the conventional dose of 50 mg/kg/day (not exceeding 3 g/d) for >3 days. Patients in critical conditions were given double doses (100 mg/kg/d) for the first 1–2 days. Thiabendazole was tapered off when clinical improvement or disappearance of larvae in feces was confirmed.

After 2003, all patients were treated with ivermectin at the dose of 200 µg/kg/day. Two patients with critical conditions received 2 or 1.5 times the conventional dose. Ivermectin was administered either 1–2 weeks apart or consecutively until the confirmation of clinical improvement or disappearance of larvae from feces. A combination of oral and rectal forms of ivermectin was administered in 3 patients.

One patient in 1980 initially received pyrvinium pamoate at a dose of 20 mL/day because thiabendazole was not available. Another patient with occult dissemination with culture-negative suppurative meningitis did not receive anthelmintics because meningitis was suspected to be caused by the central nervous system involvement of adult T-cell leukemia.

## Discussion

Disseminated strongyloidiasis is a medical emergency. Because of its rarity, most previous reports consist of single cases (*24*), making it difficult to determine its clinical phenotypes. Previous reports identified atypical suppurative meningitis, defined as community-acquired meningitis caused by enteric organisms or culture-negative suppurative meningitis, as a form of occult dissemination (*10*,*14*–*16*). Our study corroborates preceding analyses and extends their findings by demonstrating that early recognition of occult dissemination is lifesaving.

Demographic characteristics in our study, such as age distribution and male-to-female ratio, were in keeping with previous reports (*24*). However, our patients all were born before 1964, and the number of annually reported cases was decreasing during the study period. These findings were consistent with a report from another institution in Okinawa (*25*) and suggest that patients, predominantly men, in this region in the past became infected from contaminated soil during farming and by walking barefoot (*26*). Improved farming environments and changes in lifestyle might have reduced the risk for exposure. Another trend we noted was that 9 of 11 reported cases during the last 10 years of the study period were classified as dissemination and only 2 out of 11 as occult dissemination. The median age of patients in these 11 cases was 80 years, implying that the elderly who had been exposed to *S. stercoralis* parasites in their youth developed systemic strongyloidiasis at advanced age, which quickly disseminated because of their impaired immune status.

In this case series, few patients had risk factors for strongyloidiasis other than HTLV-1 infection, including recent steroid use (*3*), organ transplant (*4*), or cirrhosis (*27*,*28*). In areas with HTLV-1 endemicity, HTLV-1 infection is the major risk factor for both chronic strongyloidiasis and dissemination (*29*). Although diabetes mellitus has been reported to be a risk factor for strongyloidiasis treatment failure (*30*), the prevalence in our series remained low.

Considering the mortality rates and the various symptoms and clinical diagnoses, dissemination is the most severe form of strongyloidiasis, followed by occult dissemination with meningitis caused by enteric organisms and then occult dissemination with culture-negative suppurative meningitis. Evidence of multiorgan involvement was less common in occult dissemination, which might reflect the degree of involvement of larvae and enteric organisms in these 3 phenotypes. Previous studies have identified different degrees of their involvement (*11*,*14*,*15*) (Table 4).

**Table 4:** Involvement of larvae and enteric organisms in disseminated strongyloidiasis reported in previous studies*

Characteristic	Dissemination	Occult dissemination with meningitis/enteric organisms	Occult dissemination with culture-negative suppurative meningitis
Involvement of larvae	Confirmed (rhabditiform and filariform larvae)	Confirmed (rhabditiform larvae)	Confirmed (rhabditiform larvae)
Involvement of enteric organisms	Confirmed	Confirmed	Not detected

*References *11*, *14*, and *15*.

Despite the low mortality rate associated with occult dissemination with culture-negative suppurative meningitis, it is critical for clinicians to identify concomitant strongyloidiasis because patients might later have recurrent meningitis in more critical conditions (*18*) if not properly treated. Culture-negative suppurative meningitis should prompt the immediate consideration of performing a parasitologic examination in areas where strongyloidiasis and HTLV-1 are endemic. Previous reports have suggested that culture-negative suppurative meningitis is a form of dissemination (*10*,*15*,*17*).

Limited data are available about the optimal treatment of dissemination. Treatment options consist of reducing immunosuppressive therapies and initiating antihelmintics and antibiotics. Reducing immunosuppressive therapies is recommended because immunosuppressive therapies can induce dissemination (*6*). In a systematic review comparing ivermectin, albendazole, and thiabendazole, ivermectin resulted in better parasitologic cure than albendazole and fewer adverse effects than thiabendazole (*31*). The conventional dose of ivermectin is 200 μg/kg/day orally for 2 days, either consecutively or 2 weeks apart (*32*). In dissemination, the dose and duration are determined clinically. Repeated or prolonged administration are preferred until patients respond or until larvae are no longer detected (*33*). For patients who are unable to take oral ivermectin, a rectal (*34*), veterinary subcutaneous (*35*), or parenteral (*36*) formula is administered. The use of veterinary ivermectin is considered in critical cases. Some studies have also reported the use of a combination of ivermectin and albendazole (*37*). In our study, several critical patients received an increased dose of thiabendazole or ivermectin. No patients received a combination of ivermectin and thiabendazole. Anthelmintic agents were continued until patients responded or until larvae disappeared.

Initiation of antibiotics that target enteric organisms is essential. The antibiotics must penetrate into the central nervous system when meningitis is suspected. The spectrum should be based on the local antibiogram. In our study, no patients had bacterial infection that produced extended spectrum β-lactamases or AmpC β-lactamases. Clinicians should also notice that the results of blood and CSF cultures differed in 4 patients (4/70 [5.7%]), suggesting that larvae might have carried multiple enteric organisms. Empiric antibiotics that sufficiently cover most enteric organisms, such as third-generation cephalosporins, are recommended. The administration of dexamethasone for bacterial meningitis should carefully be considered because it might induce dissemination (*9*).

Our investigation has several limitations. First, the retrospective nature of this study might have resulted in distortions in the accuracy of the retrieved information. Second, patients with disseminated strongyloidiasis have also been admitted to other medical centers in Okinawa (*38*); hence, we might have underreported the prevalence of dissemination in this region. Third, the findings cannot be generalizable to regions where HTLV-1 infection is not endemic.

In summary, we classified disseminated strongyloidiasis into 3 phenotypes on the basis of their severity and various symptoms: dissemination, occult dissemination with meningitis caused by enteric organisms, and occult dissemination with culture-negative suppurative meningitis. Early identification of occult dissemination is lifesaving. Treatment options consist of reduction of immunosuppressive therapies, initiation of anthelmintic agents with a higher dose and longer duration depending on the severity of symptoms, and concurrent use of antimicrobial agents against enteric organisms.

## References

[1] Olsen A, van Lieshout L, Marti H, Polderman T, Polman K, Steinmann P, et al. Strongyloidiasis—the most neglected of the neglected tropical diseases? Trans R Soc Trop Med Hyg. 2009;103:967–72. 10.1016/j.trstmh.2009.02.01319328508

[2] Igra-Siegman Y, Kapila R, Sen P, Kaminski ZC, Louria DB. Syndrome of hyperinfection with *Strongyloides stercoralis.* Rev Infect Dis. 1981;3:397–407. 10.1093/clinids/3.3.3977025145

[3] Fardet L, Généreau T, Poirot JL, Guidet B, Kettaneh A, Cabane J. Severe strongyloidiasis in corticosteroid-treated patients: case series and literature review. J Infect. 2007;54:18–27. 10.1016/j.jinf.2006.01.01616533536

[4] Marty FM. Strongyloides hyperinfection syndrome and transplantation: a preventable, frequently fatal infection. Transpl Infect Dis. 2009;11:97–9. 10.1111/j.1399-3062.2009.00383.x19400809

[5] Sánchez PR, Guzman AP, Guillen SM, Adell RI, Estruch AM, Gonzalo IN, et al. Endemic strongyloidiasis on the Spanish Mediterranean coast. QJM. 2001;94:357–63. 10.1093/qjmed/94.7.35711435631

[6] Keiser PB, Nutman TB. *Strongyloides stercoralis* in the immunocompromised population. Clin Microbiol Rev. 2004;17:208–17. 10.1128/CMR.17.1.208-217.200414726461PMC321465

[7] US Centers for Disease Control and Prevention. Parasites: *Strongyloides*. Biology [cited 2019 Feb 1]. https://www.cdc.gov/parasites/strongyloides/biology.html

[8] Mansfield LS, Alavi A, Wortman JA, Schad GA. Gamma camera scintigraphy for direct visualization of larval migration in *Strongyloides stercoralis-*infected dogs. Am J Trop Med Hyg. 1995;52:236–40. 10.4269/ajtmh.1995.52.2367694965

[9] Pukkila-Worley R, Nardi V, Branda JA. Case records of the Massachusetts General Hospital. Case 28-2014. A 39-year-old man with a rash, headache, fever, nausea, and photophobia. N Engl J Med. 2014;371:1051–60. 10.1056/NEJMcpc140588625207769

[10] Kishaba T. Disseminated strongyloidiasis. In: Shiroma Y, Sato Y, editors. *Strongyloides stercoralis* and strongyloidiasis in Japan [in Japanese]. Fukuoka (Japan): Kyushu University Press; 1997. p. 55–78.

[11] Kishaba T. Strongyloidiasis and infections due to enteric organisms: sepsis, pneumonia and meningitis caused by Gram-negative bacteria associated with disseminated strongyloidiasis [in Japanese]. Infection. 1982;12:180.

[12] Jain AK, Agarwal SK, el-Sadr W. *Streptococcus bovis* bacteremia and meningitis associated with *Strongyloides stercoralis* colitis in a patient infected with human immunodeficiency virus. Clin Infect Dis. 1994;18:253–4. 10.1093/clinids/18.2.2538161638

[13] Chu E, Whitlock WL, Dietrich RA. Pulmonary hyperinfection syndrome with *Strongyloides stercoralis.* Chest. 1990;97:1475–7. 10.1378/chest.97.6.14752347234

[14] Kishaba T, Uchihara T, Ueno K, Goeku C, Shimabukuro Y. Gram-negative rod meningitis probably caused by occult disseminated strongyloidiasis [in Japanese]. Okinawa Medical Journal. 1985;22:3.

[15] Kishaba T, Suginohara K, Tamaki K, Miyara Y, Endo K, Taira Y, et al. Culture negative suppurative meningitis probably caused by occult disseminated strongyloidiasis [in Japanese]. Okinawa Medical Journal. 1989;26:219.

[16] Mukaigawara M, Nakayama I, Gibo K. Strongyloidiasis and culture-negative suppurative meningitis, Japan, 1993–2015. Emerg Infect Dis. 2018;24:2378–80. 10.3201/eid2412.18037530457540PMC6256405

[17] Sasaki Y, Taniguchi T, Kinjo M, McGill RL, McGill AT, Tsuha S, et al. Meningitis associated with strongyloidiasis in an area endemic for strongyloidiasis and human T-lymphotropic virus-1: a single-center experience in Japan between 1990 and 2010. Infection. 2013;41:1189–93. 10.1007/s15010-013-0483-223761268PMC3832757

[18] Shimasaki T, Chung H, Shiiki S. Five cases of recurrent meningitis associated with chronic strongyloidiasis. Am J Trop Med Hyg. 2015;92:601–4. 10.4269/ajtmh.14-056425548379PMC4350558

[19] Toma H, Shimabukuro I, Kobayashi J, Tasaki T, Takara M, Sato Y. Community control studies on *Strongyloides* infection in a model island of Okinawa, Japan. Southeast Asian J Trop Med Public Health. 2000;31:383–7.11127344

[20] Sagara Y, Iwanaga M, Morita M, Sagara Y, Nakamura H, Hirayama H, et al. Fine-scale geographic clustering pattern of human T-cell leukemia virus type 1 infection among blood donors in Kyushu-Okinawa, Japan. J Med Virol. 2018;90:1658–65. 10.1002/jmv.2523929900555PMC6120527

[21] Cresci GA, Bawden E. Gut microbiome: what we do and don’t know. Nutr Clin Pract. 2015;30:734–46. 10.1177/088453361560989926449893PMC4838018

[22] Scowden EB, Schaffner W, Stone WJ. Overwhelming strongyloidiasis: an unappreciated opportunistic infection. Medicine (Baltimore). 1978;57:527–44. 10.1097/00005792-197811000-00004362122

[23] Tomori M, Mukaigawara M, Narita M. Acute esophageal necrosis associated with *Strongyloides stercoralis* hyperinfection. Am J Trop Med Hyg. 2019;100:1037–8. 10.4269/ajtmh.18-066431088606PMC6493964

[24] Geri G, Rabbat A, Mayaux J, Zafrani L, Chalumeau-Lemoine L, Guidet B, et al. *Strongyloides stercoralis* hyperinfection syndrome: a case series and a review of the literature. Infection. 2015;43:691–8. 10.1007/s15010-015-0799-126008854

[25] Tanaka T, Hirata T, Parrott G, Higashiarakawa M, Kinjo T, Kinjo T, et al. Relationship among *Strongyloides stercoralis* infection, human T-cell lymphotropic virus type 1 infection, and cancer: a 24-year cohort inpatient study in Okinawa, Japan. Am J Trop Med Hyg. 2016;94:365–70. 10.4269/ajtmh.15-055626621566PMC4751948

[26] Schär F, Trostdorf U, Giardina F, Khieu V, Muth S, Marti H, et al. *Strongyloides stercoralis*: global distribution and risk factors. PLoS Negl Trop Dis. 2013;7:e2288. 10.1371/journal.pntd.000228823875033PMC3708837

[27] Teixeira MC, Pacheco FT, Souza JN, Silva ML, Inês EJ, Soares NM. *Strongyloides stercoralis* infection in alcoholic patients. BioMed Res Int. 2016;2016:4872473. 10.1155/2016/487247328105424PMC5220430

[28] de Oliveira LC, Ribeiro CT, Mendes DM, Oliveira TC, Costa-Cruz JM. Frequency of *Strongyloides stercoralis* infection in alcoholics. Mem Inst Oswaldo Cruz. 2002;97:119–21. 10.1590/S0074-0276200200010002111992161

[29] Gotuzzo E, Terashima A, Alvarez H, Tello R, Infante R, Watts DM, et al. *Strongyloides stercoralis* hyperinfection associated with human T cell lymphotropic virus type-1 infection in Peru. Am J Trop Med Hyg. 1999;60:146–9. 10.4269/ajtmh.1999.60.1469988339

[30] Hays R, Esterman A, McDermott R. Type 2 diabetes mellitus is associated with *Strongyloides stercoralis* treatment failure in Australian aboriginals. PLoS Negl Trop Dis. 2015;9:e0003976. 10.1371/journal.pntd.000397626295162PMC4546619

[31] Henriquez-Camacho C, Gotuzzo E, Echevarria J, White AC Jr, Terashima A, Samalvides F, et al. Ivermectin versus albendazole or thiabendazole for *Strongyloides stercoralis* infection. Cochrane Database Syst Rev. 2016; (1):CD007745. 10.1002/14651858.CD007745.pub326778150PMC4916931

[32] Zaha O, Hirata T, Kinjo F, Saito A, Fukuhara H. Efficacy of ivermectin for chronic strongyloidiasis: two single doses given 2 weeks apart. J Infect Chemother. 2002;8:94–8. 10.1007/s10156020001311957127

[33] Mejia R, Nutman TB. Screening, prevention, and treatment for hyperinfection syndrome and disseminated infections caused by *Strongyloides stercoralis.* Curr Opin Infect Dis. 2012;25:458–63. 10.1097/QCO.0b013e3283551dbd22691685PMC3430846

[34] Tarr PE, Miele PS, Peregoy KS, Smith MA, Neva FA, Lucey DR. Case report: Rectal adminstration of ivermectin to a patient with *Strongyloides* hyperinfection syndrome. Am J Trop Med Hyg. 2003;68:453–5. 10.4269/ajtmh.2003.68.45312875295

[35] Barrett J, Broderick C, Soulsby H, Wade P, Newsholme W. Subcutaneous ivermectin use in the treatment of severe *Strongyloides stercoralis* infection: two case reports and a discussion of the literature. J Antimicrob Chemother. 2016;71:220–5. 10.1093/jac/dkv31526462990

[36] Marty FM, Lowry CM, Rodriguez M, Milner DA, Pieciak WS, Sinha A, et al. Treatment of human disseminated strongyloidiasis with a parenteral veterinary formulation of ivermectin. Clin Infect Dis. 2005;41:e5–8. 10.1086/43082715937753

[37] Pornsuriyasak P, Niticharoenpong K, Sakapibunnan A. Disseminated strongyloidiasis successfully treated with extended duration ivermectin combined with albendazole: a case report of intractable strongyloidiasis. Southeast Asian J Trop Med Public Health. 2004;35:531–4.15689061

[38] Kishimoto K, Hokama A, Hirata T, Ihama Y, Nakamoto M, Kinjo N, et al. Endoscopic and histopathological study on the duodenum of *Strongyloides stercoralis* hyperinfection. World J Gastroenterol. 2008;14:1768–73. 10.3748/wjg.14.176818350608PMC2695917

# Review article

## Earning CME Credit

To obtain credit, you should first read the journal article. After reading the article, you should be able to answer the following, related, multiple-choice questions. To complete the questions (with a minimum 75% passing score) and earn continuing medical education (CME) credit, please go to http://www.medscape.org/journal/eid. Credit cannot be obtained for tests completed on paper, although you may use the worksheet below to keep a record of your answers. 

You must be a registered user on http://www.medscape.org. If you are not registered on http://www.medscape.org, please click on the “Register” link on the right hand side of the website. 

Only one answer is correct for each question. Once you successfully answer all post-test questions, you will be able to view and/or print your certificate. For questions regarding this activity, contact the accredited provider, CME@medscape.net. For technical assistance, contact CME@medscape.net. American Medical Association’s Physician’s Recognition Award (AMA PRA) credits are accepted in the US as evidence of participation in CME activities. For further information on this award, please go to https://www.ama-assn.org. The AMA has determined that physicians not licensed in the US who participate in this CME activity are eligible for *AMA PRA* *Category 1 Credits™*. Through agreements that the AMA has made with agencies in some countries, AMA PRA credit may be acceptable as evidence of participation in CME activities. If you are not licensed in the US, please complete the questions online, print the AMA PRA CME credit certificate, and present it to your national medical association for review.

## Article Title:  Clinical Characteristics of Disseminated Strongyloidiasis, Japan, 1975–2017

## CME Questions

1. **Your patient is a 60-year-old man with human T-cell lymphotropic virus type 1 (HTLV-1) infection who was admitted for suspected strongyloidiasis. According to the Japanese case series by Mukaigawara and colleagues, which of the following statements about classification of disseminated strongyloidiasis into 3 clinical phenotypes is correct? **
  - Most patients had occult dissemination with meningitis caused by enteric organisms
  - Mortality was greatest among patients with occult dissemination with culture-negative suppurative meningitis
  - Sepsis occurred in 40.6% of patients with dissemination
  - The number of reported cases increased each year
2. **According to the Japanese case series by Mukaigawara and colleagues, which of the following statements about clinical and laboratory findings in disseminated strongyloidiasis is correct?**
  - Common symptoms included fever (52.9%), headache (32.9%), mental status changes (24.3%), and nausea/vomiting (14.3%)
  - Half of patients tested for HTLV-1 with serological assay had positive findings
  - Predominant clinical syndromes were sepsis caused by nonenteric organisms, heart failure, and disseminated rash
  - *Enterococcus* spp. were the most frequent isolates from blood and cerebrospinal fluid (CSF) cultures
3. **According to the Japanese case series by Mukaigawara and colleagues, which of the following statements about treatment of disseminated strongyloidiasis is correct? **
  - Before 2002, most patients were treated with pyrvinium pamoate 20 mL/d
  - After 2003, patients were treated with ivermectin, 200 μg/kg/d, either 1 to 2 weeks apart or consecutively until clinical improvement or disappearance of larvae from stool
  - Patients with occult dissemination should be observed and treated only if they deteriorate
  - Treatment should be limited to antihelmintic agents
